# Supplementary material for: Are carbon emissions trading and green financial instruments synergistic? -Comprehensive quantitative research based on content analysis
Source: PLoS One. 2024 Mar 7;19(3):e0298601. doi: 10.1371/journal.pone.0298601 (PMC10919601; doi:10.1371/journal.pone.0298601)
Supplement: S1 Data — (ZIP) [file pone.0298601.s001.zip › Data/地级市Z_F工作报告.zip!/║╙─╧/╚2├┼╧┐╩╨/└┤╫╘╣1⁄2╓┌║┼í╛┬φ┐╦╩2╛▌═°í┐_2018.docx]

各位代表：

　　现在，我代表市人民政府，向大会报告工作，请予审议，并请各位政协委员和其他列席人士提出意见。

　　一、2017年工作回顾

　　2017年是本届政府开局之年，也是极不平凡的一年。一年来，我们紧密团结在以习近平同志为核心的党中央周围，全面学习贯彻党的十九大精神，强化“四个意识”，坚定“四个自信”，在省委、省政府和市委的正确领导下，在市人大、市政协的监督支持下，团结和依靠全市人民，紧盯打造省际中心城市这一目标，聚焦产业转型、打造“五彩三门峡”，发力“三次创业”、建设“三地五中心”，锐意进取，担当有为，圆满完成了市七届人大一次会议确定的目标任务，取得了好于计划、好于同行、好于预期的可贵成绩。

　　经济运行稳中向好。全市生产总值完成1460.8亿元，同比增长8.5%，增速居全省第2位；财政一般公共预算收入108.2亿元，增长10.7%；规上工业增加值622.5亿元，增长9.1%，增速居全省第1位；固定资产投资1976.9亿元，增长11.5%；社会消费品零售总额493.9亿元，增长12%；城镇居民人均可支配收入27562元，增长9.1%，农村居民人均可支配收入13084元，增长9.2%。9项主要经济指标中有5项增速高于全省平均水平，生产总值、规上工业增加值、城镇居民收入等指标迈入全省第一方阵。

　　发展质量持续提升。经济增速与发展质量同步提高，税收占一般公共预算收入比重达到70.3%，高于全省1.7个百分点。居民收入与生产总值同步增长，城乡居民人均可支配收入增速分别比上年提高3.1和1.1个百分点，均高于GDP增速。生态环境质量与经济质量效益同步改善，全市空气质量优良天数244天，居全省第3位，在全国考评中晋升25个位次，晋位幅度居全省第1位； PM10、PM2.5累计平均浓度分别居全省第4位、第5位，成为三项指标全面完成省定目标的两个地级市之一。社会事业与经济发展同步提升，三门峡社会管理学院、三门峡技师学院当年获批、当年招生，成功承办大健康产业联盟大会，仰韶村考古遗址公园和庙底沟考古遗址公园获批国家级遗址公园，2台剧目获“河南文华大奖”。

　　重大攻坚扎实推进。金融扶贫走在全国前列，全国、全省金融扶贫现场会在我市召开，卢氏模式、陕州经验在全国推广。去产能攻坚、省属国有企业“三供一业”剥离，以及国企改革“僵尸企业”处置工作均走在全省前列。环境治理攻坚成效明显，小秦岭自然保护区在“绿盾2017”国家监督检查专项行动中获得第一名。三门峡进口铜精矿国检试验区顺利获批，国家果品与果蔬汁检测重点实验室通过验收。

　　重大项目实现突破。速达10万辆电动汽车项目获国家发改委批准，获得河南首张、全国首批新能源汽车项目牌照，打造新能源汽车产业集群迈出关键性步伐。国道310南移项目全面开工，完成年度投资目标的104%。三门峡黄金硅谷城挂牌运营。

　　重大改革成效显著。市投资集团投入运营，初步形成了超百亿元的融资能力，为经济建设注入了强劲动力。新型智慧城市“一平台、两中心、三应用”项目建成投用，互联网+政务服务、“12345”便民服务热线、城市运营指挥中心投入运营，全省领先的新型智慧城市基本框架初步形成。三门峡产业集聚区增量配电业务改革试点获得国家批复。

　　重大活动圆满成功。作为河南省唯一入选城市参加央视首届“魅力中国城”活动，荣获“十佳魅力城市”称号，当选“2017中国年度文化影响力城市十强”，全市接待海内外游客数、旅游总收入分别增长14.5%和16.8%，“黄河三门峡·魅力天鹅城”城市品牌叫响全国。

　　一年来，我们主要抓了以下工作：

　　（一）坚持稳中求进工作总基调，经济发展迈出新步伐。坚持把推动经济平稳增长作为全局工作的首要任务，全力以赴稳增长、保态势、防风险。着力扩大投资。全市19个省重点项目完成投资121.5亿元，占年度目标的107%；240个市重点项目完成投资774亿元，占年度目标的133%。戴卡轮毂等92个项目建成投产，中金二期等123个新建项目开工，同人铝业铝精深加工项目复工，为转型发展奠定了坚实基础。着力强化保障。坚持分类指导，盘活处置61家停产半停产企业，有力推动了工业经济平稳运行。积极组织银企对接，签约总金额达417亿元。着力化解风险。加大打击和处置非法集资工作力度，10起案件进入兑付阶段，最大限度挽回了群众利益，守住了不发生区域性金融风险的底线。

　　（二）坚持问题导向破解发展难题，重大攻坚获得新成果。落实省委、省政府战略部署，成立专门督查巡查机构，全力打好四大攻坚战。强力推进脱贫攻坚。实施产业扶贫“百千万”工程，实现2.76万人脱贫、1.15万人易地搬迁脱贫，超额完成省定目标，去年上半年核查评估中居全省第2位。党建扶贫、金融扶贫、健康保险扶贫等工作走在全省乃至全国前列。持续推进环境治理攻坚。大气污染防治扎实有效，空气质量大幅好转。水污染防治全面启动，推行四级“河长制”，7个省定河流断面年均值达标率100%，水环境质量明显改善。扎实开展生态环境治理，黄河湿地国家级自然保护区生态治理任务全面完成。

　　扎实开展国企改革攻坚。成立市政府国资委，全面理清市属国有企业资产底数，初步摸排62家市属国有“僵尸企业”并建立台账，完成16家企业处置工作。剥离省属国有企业职工“三供一业”4.9万户，移交退休人员2万人，接收转移社保8.3万人，完成任务量居全省第3位，被评为全省国企改革攻坚战先进市。深入实施转型发展攻坚。强力推动工业转型，制定实施《三门峡市工业转型升级考核办法》《三门峡市“巨人”企业培育计划》，高新技术产业、高成长性制造业增加值分别增长12.9%和16.2%，对工业的贡献率分别达到23.5%和20.7%。

　　（三）坚持深化供给侧结构性改革，结构升级取得新进展。扎实推进 “去降补”任务落实。完成14处煤矿、240万吨煤炭产能退出任务，关闭矿井数和去产能总量居全省第4位；房地产去化周期降至7个月；重点企业“债转股”工作稳步推进；十大领域累计减轻实体经济负担8.3亿元。加快产业结构优化升级。实施亿元以上工业转型升级项目55个，完成投资275.2亿元，义腾新能源等20个项目建成投产。第三产业占GDP的比重达到35.1%，较上年提高0.9个百分点，对经济增长的贡献率达到36.8%。涉农电商交易额突破25亿元。注重品牌带动。“骏通”“二仙坡”荣获中国驰名商标称号。新认定“三品一标”农产品14个，“灵宝香菇”获得全省首个国家级农产品地理标志示范样板，“灵宝苹果”荣获“年度魅力品牌”称号。

　　（四）坚持贯彻落实新发展理念，创新驱动凝聚新动力。坚持改革激发内生动力。积极创新投融资模式，产业基金、PPP项目等工作扎实推进。“放管服”改革、“效能革命”、“最多跑一次”改革持续深化；商事制度改革成效显著，受到国务院表彰；营改增、医药卫生、金融、价格、电力、盐业等改革取得新进展。坚持开放增强外源动力。大力开展招商引资，引进签约项目87个，总投资591亿元。央企合作扎实推进，成功引进航天科工、国投、中建、中青旅等24家央企，签订合作项目25个，已开工17个，总投资414亿元。区域合作迈出新步伐，三门峡—平陆城际公交开通，晋陕豫商标受理窗口在我市设立。与河南科技大学等高校的校地合作积极推进。坚持创新培育新动能。出台《关于支持企业创新的实施意见》，科技创新政策激励机制不断完善。16家企业被认定为国家高新技术企业，三门峡中原精密有限公司等6家企业入选省“科技小巨人培育企业”，新认定省级工程技术研究中心4家，新认定国家级星创天地1家、省级3家，全市各类企业研发平台达到149个，科技创新能力稳步提升。

　　（五）坚持城乡区域协调发展，新型城镇化呈现新气象。《百年崤函——三门峡2057远景战略发展规划》和《三门峡市中心城区建设提质规划》编制全面启动，全市城镇化率54.7%，居全省第6位。持续提升中心城区品位。文明城市创建暨百城建设提质工程稳步推进。城市新增供热面积210万平方米，大岭路等13条道路升级改造完成，北环路等5条断头路开工，上村、崖底等旧城区和城中村改造加快实施，22个棚改项目全部开工，是近年来市政基础设施建设力度最大、成效最大的一年。商务中心区完成投资超百亿元，基础设施和公共服务不断完善，正在成为带动中心城区发展的增长极。持续增强城镇辐射带动能力。市、县城区基础设施扩容提质稳步推进，市城乡一体化示范区管理体制套合完成，全市7个国家级重点镇和灵宝市函谷关特色小镇的综合发展能力、人口承载能力和经济实力不断提升，辐射带动作用不断增强。持续推动美丽乡村建设。农村人居环境持续提升，基础设施不断完善，新型农村社区遗留问题整改工作全面完成，综合排名全省第3位。

　　（六）坚持以人民为中心的发展思想，民生保障实现新进步。扎实推进就业创业。实施更加积极的就业政策，累计发放创业担保贷款4.8亿元，全市城镇新增就业4.96万人，城镇登记失业率2.76%，继续保持“零就业”家庭动态为零。兜牢民生保障底线。困难群众大病补充医保、困难残疾人两项补贴等政策全面落实，城乡低保标准持续提高，社会保障体系更加完善。建成保障房2.6万套，完成目标任务的483%。稳步发展社会事业。持续推进基础教育，新改扩建中小学15所、幼儿园16所。加快“健康三门峡”建设，出台《健康三门峡2030规划纲要》，通过河南省城市公立医院综合改革综合考评，黄河医院商务中心区分院、大健康服务中心等项目进展顺利。成功举办市八运会暨全民健身大会。深入推进社会主义核心价值观建设，群众性精神文明创建活动蓬勃开展。社会大局保持稳定。扎实开展安全生产和信访稳定攻坚，安全生产形势总体平稳，信访工作走在全省前列。

　　一年来，我们始终贯彻全面从严治党要求，全力推进“两学一做”学习教育常态化制度化，牢记宗旨，勤勉尽责，努力为人民提供优质高效服务。自觉接受人大法律监督、政协民主监督和社会监督，认真办理人大代表建议227件、政协委员提案268件。加强民主法治建设，全面推进依法行政，不断强化行政监察和审计监督，加强与民主党派、工商联、无党派人士及各人民团体的联系，政府自身建设和治理能力不断提升。严格落实“一岗双责”，驰而不息正风肃纪，坚定不移惩治腐败，廉洁政府建设取得新成效。加强国防教育、国防后备力量和人民防空建设，推进军民融合深度发展，驻三门峡解放军、武警部队、预备役人员、民兵为地方发展作出新贡献，双拥工作持续深化。统计、审计、外侨、民族宗教、对台、史志、气象、防震减灾、无线电、黄河河务、妇女儿童、残疾人、老龄、慈善等工作取得新成绩。

　　各位代表！本届政府工作的良好开局来之不易，这是在习近平新时代中国特色社会主义思想的指引下，省委、省政府和市委正确领导的结果，是全市人民凝心聚力、拼搏奋斗的结果。在此，我代表市人民政府，向全市各行各业的劳动者，向各位人大代表、政协委员，向各民主党派和人民团体，向驻三门峡解放军、武警官兵、公安民警，向广大客商以及关心支持三门峡发展的各界人士，致以崇高的敬意和衷心的感谢！

　　我们也清醒认识到，经济社会发展不平衡不充分问题还比较突出，工作还存在不少挑战和薄弱环节。主要表现为：一是产业结构不优，层次偏低，服务业占比分别低于全省、全国7.6个和16.5个百分点，传统产业占工业增加值比重近七成，新兴产业还处于初期培育阶段，新动能支撑不力，转型发展任重道远；二是新型城镇化质量不高，城市建设品质不优，城乡发展不平衡不充分问题较多，全市还有8.8万未脱贫人口，统筹城乡发展任务繁重；三是创新发展水平不足，2016年全市研发经费占生产总值的比重只有0.63%，仅相当于全省的一半；四是基础支撑能力不强，交通、能源、水利、生态等基础设施建设和相关保障水平仍不到位，支撑发展的高层次人才不足；五是民生领域仍有短板，就业、教育、卫生、住房等公共服务水平不高，安全生产、公共安全等领域依然存在薄弱环节。同时，政府职能转变还不到位，一些政府工作人员综合素质不能适应新时代要求，“四风”问题仍然存在。对此，我们一定勇于担当，切实解决，不负人民重托。

　　二、2018年工作主要目标和重点任务

　　2018年，是贯彻党的十九大精神的开局之年，是改革开放四十周年，是决胜全面小康社会、实施“十三五”规划承上启下的关键一年。做好今年政府工作的指导思想是：全面贯彻落实党的十九大和中央、省委、市委经济工作会议精神，以习近平新时代中国特色社会主义思想为指导，持续落实习近平总书记对河南提出的“打好四张牌”和省委对三门峡提出的“四点要求”，坚持稳中求进工作总基调，坚持新发展理念，紧扣社会主要矛盾变化，按照高质量发展的要求，统筹推进“五位一体”总体布局和协调推进“四个全面”战略布局，坚持以转型创新发展为方向，以供给侧结构性改革为主线，以市委、市政府深入贯彻落实习近平总书记重要讲话精神着力打好“四张牌”五年行动计划为主题，以“效能革命”为动力源泉，着力发挥优势打造“五彩三门峡”、建设“三地五中心”，大力推动质量变革、效率变革、动力变革，坚决打好防范化解重大风险、精准脱贫、污染防治三大攻坚战，统筹推进稳增长、促改革、调结构、惠民生、防风险各项工作，谱写三门峡高质量发展的新篇章。

　　经济社会发展的主要预期目标是：地区生产总值增长8%左右，其中一产增长4%，规上工业增加值增长8.5%，三产增长9.5%；全社会固定资产投资增长9%，社会消费品零售总额增长11%左右，进出口总值保持稳定，财政一般公共预算收入增长8%以上，税收占一般公共预算收入比重70%以上，城镇居民人均可支配收入增长8.5%，农村居民人均可支配收入增长8%；城镇新增就业4万人以上，城镇登记失业率控制在4.5%以内，研发经费占生产总值的比重进一步提高，节能减排、环境保护等约束性指标完成省下达任务，供给侧结构性改革取得实质性进展，发展质量效益持续提高，人民群众获得感不断增强。

　　实现以上目标，重点抓好以下六个方面的工作：

　　（一）强力推进三大攻坚，为全面建成小康社会奠定坚实基础。全面贯彻落实中央、省决策部署，坚决打好精准脱贫、污染治理、防范化解重大风险三大攻坚战，补齐全面建成小康社会短板，确保经济社会发展行稳致远。

　　打好精准脱贫攻坚战。坚持“尽锐出战，精准施策”，强化责任落实、政策落实、工作落实，加快推动“五个融合”，确保5.3万人稳定脱贫。聚焦深度贫困。以卢氏县和232个深度贫困村为重点，整合政策资源，因村因户施策，实施教育、医疗、水利、交通、电网等五大专项方案，改善发展环境和生产条件。按照“政府主导、资源整合、市场运作、产业支撑”的原则，通过保贷统合，确保“四类”深度贫困户实现“死钱变活钱、输血变造血、保底变保富”。统筹做好非贫困县、非贫困村的精准帮扶工作，确保脱贫攻坚均衡推进。抓好重点难点。落实搬迁规划，严格掌握标准，及时拨付资金，抓紧推进项目，全面完成易地扶贫搬迁任务。围绕特色产业，大力实施产业扶贫基地“百千万”工程，实现贫困人口产业发展项目全覆盖，支持新型经营主体和贫困户建立稳定的利益联结机制，让更多贫困户在产业发展中得到实惠。持续推广卢氏模式及陕州经验，抓好金融扶贫工作。激发基层活力和内生动力。充分发挥乡、村两级在脱贫攻坚中的主体作用，进一步调动基层干部干事创业的积极性、主动性、创造性，夯实脱贫攻坚一线的“堡垒”，带动一方群众脱贫致富。把扶贫和扶志、扶智结合起来，把救急纾困和内生脱贫结合起来，把发展短平快项目和培育特色产业结合起来，改进帮扶方式方法，建立正向激励机制，变输血为造血，实现可持续稳定脱贫。严格督导考核。统筹整合各方面督查力量，实行最严格的督查巡查和考核评估，定期通报，年终总评，严格兑现奖惩。开展扶贫领域腐败和作风问题专项治理，确保脱贫工作务实、脱贫过程扎实、脱贫结果真实。

　　打好污染防治攻坚战。继续坚持全民治污、科学治污、依法治污，持续抓好大气、水、土壤污染防治，努力解决环境突出问题，不断满足人民群众日益增长的良好生态环境需要。加力推进“蓝天工程”。坚持标本兼治，突出重点区域、重点企业、重点时段，狠抓“三治标、三治本”措施落实，加快产业、能源结构调整，大力发展城市公共交通，稳步推进清洁取暖，努力让人民享有更多蓝天白云。深入实施“碧水工程”。强化四级“河长”责任，“一河一策”制定专项治理方案，系统推进水污染防治、水资源保护和生态水系建设，确保弘农涧河、枣乡河、阳平河、文峪河等重点河流全部治理到位，城市建成区实现雨污分流，污水直排全部截流到位，黑臭水体基本消除，让我们的城市充满灵性。全面启动 “净土工程”。大力推进土壤污染防治，扎实做好土壤污染状况详查，加快推进重点地区重金属污染物总量减排，严格危险废物环境监管，开展土壤污染治理与修复，努力守护一方净土。持续开展“生态修复工程”。科学划定生态保护红线和城镇开发边界控制线。大力开展国土绿化行动，完成森林抚育和改造54.6万亩，积极创建省级生态县。加强自然保护区监管，全面完成小秦岭和黄河湿地自然保护区生态修复任务。

　　打好防范化解重大风险攻坚战。强化底线思维，坚持防范化解并重，牢牢守住不发生区域性系统风险的底线。稳妥化解企业风险。按照多兼并重组、少破产关闭，多联合作战、少单兵突围的基本思路，稳步化解企业风险。积极推进市场化“债转股”，引导风险企业采取资产债务重组、存量盘活等措施解决资金危机。加快推进企业上市工作，未来2到3年内再推动10家企业挂牌上市。落实政策措施，优化营商环境，支持民营企业发展。防控政府债务风险。严控政府债务增量，科学制定化解防范方案，确保政府债务总体安全可控。根据地区承受能力，科学谋划PPP项目。防控金融风险。完善金融风险监测预警体系，稳妥有效处置非法集资，防范新的案件发生。加强信用体系建设和社会公众诚信教育，营造良好金融生态环境。

　　（二）打好产业结构优化升级牌，提高经济发展质量效益。按照“技术水平调高、制造能力调强、产业结构调优、产业链条调长”的发展方向，培育发展新技术、新产业、新业态、新模式，全面推进制造业、服务业、种养业供给侧结构性改革，把上游优势转化为终端优势，把传统产业优势转化为新兴产业优势，推动产业向中高端迈进。

　　以工业为主导，加快发展先进制造业。坚持工业强市核心战略，抓住国家“制造业三年行动”和技术改造升级工程机遇，用好优惠政策，推动黄金、铝、煤及煤化工等传统产业延链强链补链，向价值链、利润链、效益链的高端迈进，实现提升发展。大力运用新技术、新业态、新模式，加快制造业数字化、网络化、智能化、绿色化改造，推动装备制造、新能源汽车、新型材料、生物医药、食品加工等战略新兴产业扩量提质，实现创新发展，打造经济增长新引擎。

　　以服务业为引领，加快发展高成长性服务业。坚持服务业优先战略，改造提升商贸流通、房地产等传统服务业，积极发展电子商务、现代物流、现代金融、信息服务等先导产业，促进生产性服务业向专业化和价值链高端延伸，为先进制造业发展提供配套和保障。突出消费引领，围绕消费市场个性化、定制化需求，加快发展旅游、文化、体育、健康、养老五大幸福产业，促进生活性服务业向精细化和高品质转变，把现代服务业打造成为新时代经济发展新支撑。

　　以农业为根本，加快发展现代种养业。充分发挥我市特色农业的基础优势和产品优势，积极适应农产品需求变化的阶段性特征，把增加优质生态农产品供给放在突出位置，聚焦“六优”产品培育，继续提升特色农业品牌影响力。利用物联网等现代信息技术改造农业生产，促进农业规模化、标准化、产业化、信息化发展。推动农村一、二、三产业融合发展，因地制宜发展“互联网+”“旅游+”“生态+”等农业新业态，谋划建设苹果小镇、香菇小镇、温泉小镇等特色小镇，实现农业从生产向生态、生活功能拓展，培育更多繁荣农村、富裕农民的新兴支柱产业。

　　以项目为支撑，扎实开展项目建设攻坚年活动。坚持项目带动战略，完善领导分包、联审联批、督导考核、月度协调等推进机制，着力抓好18个省重点项目、221个市重点项目建设，年度完成投资600亿元以上。加快推进产业集聚区提质增效升级，确保三门峡产业集聚区顺利晋升“二星”。

　　（三）打好创新驱动发展牌，不断增强发展动力活力。坚持把创新摆在发展全局的核心位置，将改革开放创新作为动能转换的主引擎，加快构建自主创新和开放创新体系，提高区域创新能力。

　　继续深化改革。以“证照分离”和网上审批为抓手，持续推进“放管服”改革，全面实行市场准入负面清单制度，完善事中事后监管，实现“双随机、一公开”监管全覆盖，切实把该放的放到位、该管的管到位，有效发挥市场配置资源作用。扎实推进国企改革攻坚，加快企业产权、组织、治理结构改革，发展混合所有制经济，完成36家国有“僵尸企业”市场出清，完成驻我市央企及市属国有企业剥离办社会职能工作，培育一批具有较强竞争力的国有骨干企业。深入推进投融资和金融体制改革，进一步支持市投资集团做大做强，确保三门峡农商银行顺利开业。继续推进“三十五证合一”、“最多跑一次”、财税体制等改革，把改革红利更多地转化为发展动力。

　　扩大开放合作。持续抓好招商引资，围绕十大重点产业，成立专职招商小组，瞄准产业链图谱谋划包装一批优质项目，着力引进战略投资者，实现黄金珠宝、铝精深加工、煤化工、特色农业、文化旅游等主导产业招商新突破。持续深化央企合作，重点跟踪25家央企38个合作意向，落实“专人专班”工作机制，办好央企合作对接系列活动，促进更多项目落地。持续扩大开放领域，加快推进自贸区同期复制区申建工作，积极申报国家级电商示范基地、全国跨境电商服务综合试点，抓好苹果、果汁和香菇出口基地建设。积极融入“一带一路”战略，推动黄金、氧化铝等企业 “走出去”，抓好境外矿产资源开发等项目。依托三门峡进口铜精矿国检试验区，加快建设家门口的海外原料基地。持续加强区域合作。深入推进黄河金三角区域基础设施、产业发展、公共服务等合作。

　　强化创新驱动。突出创新重点，依托重点产业和重点企业，围绕产业链部署创新链，补强创新链支撑产业链，重点抓好新能源汽车、黄金铝铜精深加工、煤及煤化工、先进制造业、生物医药等主导产业技术创新，积极与中国科学院、河南科技大学等高校和科研院所开展联合攻关，谋划建设工业科技创新服务中心，建立以企业为主体、市场为导向、产学研深度融合的技术创新体系。培育创新主体，充分发挥企业主力军作用，大力实施“加快培育高新技术企业行动计划”，落实好支持高新技术企业发展的扶持政策，引导企业加大研发投入，形成创新突破点。建立一批低成本、便利化、开放式的众创空间和虚拟创新社区，孵化培育“专精特新”创新型小微企业，全年新增高新技术企业5家，科技型中小企业达到120家。引进创新人才，继续实施“崤函人才计划”，加快引进一批科技领军人才和创新团队，强化创新驱动的智力支撑。加强企业家队伍建设，大力弘扬企业家精神，在全市上下营造尊重企业家价值、鼓励企业家创新、关心企业家成长的浓厚氛围。

　　（四）打好新型城镇化牌，推动城乡融合发展。坚持以人为核心，科学推进新型城镇化，大力实施乡村振兴战略，统筹推进城乡区域融合发展。

　　强力推进新型智慧城市建设。实施网络基础设施提升工程，推动电子政务外网市县乡三级全覆盖，汇聚整合政务大数据，构建市县一体化的视联网、物联网、时空云管控平台，有序推进污染防治、安全生产、精准脱贫、便民服务等平台建设，推广智慧成果应用，增强智慧城市对重点工作、民生保障的服务和支撑能力，抢占管理、服务和发展制高点。

　　统筹推进文明城市创建和百城建设提质工程。以文明创建引导百城提质，以百城提质支撑文明创建，确保创建工作今年大头落地，明年成功创建省级文明城市，2020年冲刺国家级提名城市。持续提升城市综合承载能力。加快实施中心城区提质、百城建设提质、重点镇建设“三大工程”，不断提升基础支撑力和综合承载力，夯实文明城市创建的硬件基础。持续提升市民文明素质能力。加强社会主义核心价值观教育，大力开展市民思想道德素质、诚信守法、文明风尚“三大提升”行动，积极推进文明单位、文明校园、文明家庭建设，提升城市文明软实力。持续提升公共服务能力。以解决城市“七大难”问题为抓手，持续推动公共服务均等化和城市管理精细化。大力实施“城市双修”，加快城市生态修复，按照“城区园林化、郊区森林化”思路，提升城市生态空间档次，打造“四季常绿、三季有花”的城市绿化格局；加快城市修补，积极推进交通治“乱”、市容治“脏”、生态环境治“污”、公共服务治“差”，提升城市品质。

　　实施乡村振兴战略。按照产业兴旺、生态宜居、乡风文明、治理有效、生活富裕的总要求，科学编制好乡村振兴战略规划，建立健全城乡融合发展的体制机制。深化改革激发活力。完善承包地“三权”分置制度，落实好第二轮土地承包再延长30年政策。深化农村集体产权制度改革，抓好改革试点，盘活用好闲置农房和宅基地，深入推进资源变资产、资产变股权、资金变股金、农民变股东改革。做好第三次全国土地调查工作。补齐农村发展短板。提升农村公共服务水平，鼓励和引导更多公共服务向农村延伸，加快推动教育、医疗、卫生、文化等公共服务均等化，逐步缩小城乡差距。完善农村基础设施，加快建设“四好”农村路，新改建农村公路380公里以上；启动鸡湾水库、槐扒提水二期等工程前期工作，全面实施“五小水利”工程，改善农村生产生活条件。完善农村经营体系。大力培育新型农业经营主体，发展多种形式的规模经营，做强一批精深加工型农业龙头企业，培育一批现代农业示范企业和园区，激发农业农村发展的动力和活力。加强乡村有效治理。全面落实农村人居环境整治三年行动方案，扎实推进农村畅通、环境净化、乡村绿化、村庄亮化、农村文化 “五大工程”，建设天蓝地绿山清水秀美丽乡村。深化农村精神文明创建，持续移风易俗，弘扬时代新风，丰富农民群众文化生活，为实现乡村振兴集聚精神能量。强化农村基层组织建设，打造懂农业、爱农村、爱农民的“三农”工作队伍。

　　（五）打好基础能力建设牌，加快打造“三地五中心”。充分发挥区位优势，以“三地五中心”为载体，加快各类高端生产要素集聚，培育区域核心竞争力，为打造省际中心城市提供有力支撑。先进制造业基地方面，将速达电动汽车项目作为全市转型发展的一号工程，确保6月份建成，9月份通过工信部准入。围绕装备制造产业园、珠宝首饰加工产业园、铜精深加工产业园、大数据产业园、军民融合产业基地建设，抓好同人铝业一期、凯斯曼汽车零部件、金源朝辉压延铜箔二期、宝鑫电子锂电铜箔、骏通公司专用车、航科集团大型无人机制造、中科数遥二期等项目建设。物流基地方面，加快推进锦江集团物流园项目，加快建设有色金属期货交割仓、公共型海关监管仓库，规划建设一批标准化冷链物流储配中心。积极引进第三方物流企业、总部型物流企业、物流采购、配送中心落户三门峡，构建全产业链的仓储物流服务体系。旅游目的地方面，加快发展全域旅游，整合天鹅湖公园、黄河公园、陕州地坑院、高阳山温泉等中心城区优势旅游资源，创建国家级旅游度假区。强力推进天鹅湖国家城市湿地公园创建国家5A级旅游景区。加快推进智慧旅游、休闲绿道、旅游厕所等旅游公共服务体系建设，促进工业旅游、农业旅游、乡村旅游、购物娱乐等“旅游＋”融合发展。办好第十二届中国摄影艺术节、第二十四届三门峡黄河文化旅游节·第六届中国特色商品博览交易会。大数据中心方面，深化与海康威视等知名互联网企业深度合作，建设三门峡大数据产业园，引进培育数据应用和增值服务企业5家，电子信息、智能制造企业10家，带动500名以上大数据产业从业人员，努力打造全国有影响的特色农产品、黄金行业数据中心。黄金珠宝加工交易中心方面，加快推动中金二期、金城黄金冶炼等项目，力争上半年建成投产。依托三门峡黄金硅谷城，打造集黄金交易拍卖、黄金文化创意、跨境黄金自贸区于一体的全球创新创业平台，加快黄金产业转型升级和国际化经营步伐。大健康中心方面，科学编制大健康产业发展规划，打造以“赏白天鹅、泡矿温泉、游地坑院”为核心的精品康养旅游线路，加快大健康服务中心、黄河金三角医疗养老示范区、陕州益康桃花源温泉颐养小镇、渑池金秋乐园养老服务中心等项目建设，稳步提升健康养老产业基础支撑能力。推动市中心医院、黄河医院、中医院做优做强重点专科，引入域外优质医疗资源，加快儿童医院等6个区域性医疗中心、骨关节病等4个专科诊疗中心建设，提升区域医疗水平。客运中心方面，稳步推进陇海铁路取直、运三高铁、卢洛高速前期工作，开工建设三洋铁路、卢栾高速，加快蒙华铁路、310国道南移工程、渑栾高速建设进度，确保三淅高速豫晋省界至灵宝段建成通车，争取南站长途汽车站投入运营。职教中心方面，加快推进三门峡社会管理学院二期工程，抓好三门峡社会管理学院、三门峡技师学院招生，争取招生规模扩至2800人以上。加快推进三门峡职业技术学院新校区建设，争取河南科技大学应用工程学院独立设置，积极推动黄金工业学校升格，构建高中职衔接一体化发展的职业教育格局。

　　（六）切实保障改善民生，着力增进人民福祉。坚持以人民为中心的发展思想，集中做好普惠性、基础性、兜底性民生建设，不断满足人民日益增长的美好生活需要，使人民群众有更多获得感、幸福感。持续增加居民收入。实施城乡居民收入“倍增计划”，着力抓好产业发展和改革深化，拓宽居民劳动收入和财产收入渠道，夯实增收基础；着力完善分配机制，稳步提高工资收入在家庭总收入中的比重，提高一线劳动者的报酬，适时提高社会最低工资标准，不断提高社会保险、城乡低保、特困对象等保障标准，增加低收入群体收入，扩大中等收入群体比重，确保居民收入增速高于经济增速。着力扩大社会就业。完善就业创业服务体系，深入实施全民技能振兴工程，统筹推进高校毕业生、农村转移劳动力、城镇困难人员、退役军人、去产能分流职工等重点群体就业工作。完善社会保障体系。实施全民参保计划，完成机关事业单位养老保险制度改革，整合城乡居民基本医疗保险制度，构建多层次社会保障体系。全面落实困难群众大病补充保险制度，深化以按病种付费为主的医保支付方式改革，加快推进跨省异地就医即时结算。落实差别化房地产调控政策，加快建立适合我市实际的租购并举住房制度，加快推进投资143亿元的12个棚户区改造项目。大力发展社会事业。坚持教育优先发展，完成“全面改薄”“城乡义务教育资源扩充计划”和薄弱普通高中改造任务，引进东方剑桥和上海师范大学等优质教育集团，新增6000个高质量义务教育学位，全面启动原市中专、市技校改建义务教育阶段学校项目，促进义务教育均衡优质发展。加快医疗卫生计生事业发展，深入推进分级诊疗、家庭医生签约服务和医联体建设，稳步解决群众“看病难”“看病贵”问题。全面启动“百年仰韶”文化工程，加快推进仰韶村国家考古遗址公园、庙底沟国家考古遗址公园暨博物馆建设，提高公共文化服务水平。广泛开展全民健身运动，建成城市社区“十五分钟健身圈”。推进社会治理创新。提高社会治理社会化、法治化、智能化、专业化水平，扎实开展扫黑除恶专项斗争，加强社会治安防控体系建设，强化安全生产、食品药品安全等关键领域治理，保持“打非治违”高压态势，构建隐患排除长效机制，确保实现“一个杜绝、三个持续下降”目标。健全完善重大决策社会稳定风险评估机制，推进阳光信访、责任信访、法治信访，妥善解决人民群众合理合法利益诉求，维护社会大局和谐稳定。

　　加强国防教育和国防后备力量建设，支持国防和军队改革，支持驻三门峡解放军、武警部队、预备役部队、民兵和人民防空建设，扎实推进军民融合，深入开展双拥共建，巩固军政军民团结。支持工会、共青团、妇联、科协、侨联等人民团体更好发挥桥梁纽带作用。继续做好统计、审计、外侨、对台、史志、档案、地震、民族宗教、防灾减灾等工作。

　　三、加强政府自身建设

　　新时代新形势对政府工作提出了新要求，我们必须顺应人民群众新期待，适应实践发展新要求，努力造就信念过硬、政治过硬、责任过硬、能力过硬、作风过硬的干部队伍，加快建设人民满意政府。

　　加强政治建设。坚决把维护以习近平同志为核心的党中央权威和集中统一领导作为严格政治准则和根本政治要求，强化“四个意识”，增强“四个自信”，做到“四个服从”，坚决贯彻中央方针政策，坚决落实省委、省政府和市委的部署要求。严格执行新形势下党内政治生活若干准则，完善和落实政府议事规则和决策程序。精心组织开展“不忘初心、牢记使命”主题教育，推进“两学一做”学习教育常态化制度化。

　　加强法治建设。牢固树立法治理念，严格依法履行职责，主动接受人大法律监督、工作监督和政协民主监督，密切与各民主党派、工商联和各人民团体、社会各界人士的沟通联系，认真办理人大代表建议和政协委员提案。深入推进法治政府建设，严格执行重大行政决策制度，始终坚持依宪施政、依法行政，做到严格规范公正文明执法。

　　加强作风建设。严格落实中央八项规定实施细则以及省、市实施办法，驰而不息纠正“四风”，持续深化“效能革命”，提高政府执行力。大兴狠抓落实之风，敢于担当，敢于较真，敢于碰硬，责任面前不推诿，问题面前不回避，困难面前不退缩，以逢山开路的闯劲、攻城拔寨的拼劲、抓铁有痕的韧劲，务求各项工作落到实处、取得实效。大兴调查研究之风，深入基层沾泥土、接地气、听真话，建立健全大数据支撑科学决策机制，认真解决好群众最盼、最急、最忧的问题，用新作为展示新形象。大兴干事创业之风，强化责任担当，倡导专业精神，努力增强“八种本领”，完善正向激励机制，强化失职追责惩戒，营造“想干事、能干事、干成事，不出事”的浓厚氛围。

　　加强廉政建设。认真落实全面从严治党主体责任和“一岗双责”，不断增强政治定力、纪律定力、道德定力、抵腐定力。深入推进政府系统党风廉政建设，健全行政权力监督约束机制，全面推进政务公开，强化审计监督，推动政府层级监督，主动接受舆论监督和群众监督。坚决惩治各种形式的腐败行为，坚决清除各种腐败源头，营造风清气正的政治生态。

　　各位代表！昂首阔步新时代，扬帆启航新征程。让我们更加紧密地团结在以习近平同志为核心的党中央周围，在省委、省政府和市委的坚强领导下，不忘初心、牢记使命，以更加出彩的业绩，奋力谱写三门峡高质量发展的新篇章！
